# Effect of arm position on the prediction of kinematics from EMG in amputees

**DOI:** 10.1007/s11517-012-0979-4

**Published:** 2012-10-23

**Authors:** Ning Jiang, Silvia Muceli, Bernhard Graimann, Dario Farina

**Affiliations:** 1 Department of Neurorehabilitation Engineering, Bernstein Focus Neurotechnology Göttingen, Bernstein Center for Computational Neuroscience, University Medical Center Göttingen, Von-Siebold-str. 4, 37075 Göttingen, Germany; 2 Otto Bock HealthCare GmbH, Max-Näder Straße 15, 37115 Duderstadt, Germany; 3 Center for Sensory-Motor Interaction, Department of Health Science and Technology, Aalborg University, Fredrik Bajers Vej 7D2, 9220 Aalborg, Denmark

**Keywords:** Electromyography, Myoelectric control, Kinematics estimation, Upper limb prosthesis

## Abstract

Myoelectric control has been extensively applied in multi-function hand/wrist prostheses. The performance of this type of control is however, influenced by several practical factors that still limit its clinical applicability. One of these factors is the change in arm posture during the daily use of prostheses. In this study, we investigate the effect of arm position on the performance of a simultaneous and proportional myoelectric control algorithm, both on trans-radial amputees and able-bodied subjects. The results showed that changing arm position adversely influences the performance of the algorithm for both subject groups, but that this influence is less pronounced in amputee subjects with respect to able-bodied subjects. Thus, the impact of arm posture on myoelectric control cannot be inferred from results on able-bodied subjects and should be directly investigated in amputee subjects.

## Introduction

Electromyography (EMG) has been used as the control source for powered upper limb prostheses for several decades. However, myoelectric controlled prostheses have still limited functionality since the number of reliable functions per channel pair never exceeds three [16]. Research has thus focused on pattern recognition algorithms. These methods achieve high performance (>95 % accuracy in >10 motion classes) in laboratory conditions [15]. Despite good laboratory performance, EMG pattern recognition for prosthetic control has practical limitations (see [10] for a recent review). One of the problems is that pattern recognition of the EMG does not provide simultaneous and proportional control of multiple functions, but only sequential and on/off activation. Simultaneous and proportional myoelectric control over multiple degrees of freedom (DoFs) can be achieved with alternative approaches, for example based on the synergistic structure of muscle activation [9, 13, 14]. With this method, three DoFs of the wrist [9], [14] as well as hand open/close [13] could be estimated from the EMG with good accuracy in both able-bodied subjects and trans-radial amputees.

Another problem identified when applying pattern recognition methods is that when the arm position changes with respect to the training measures, the performance drops substantially (up to 40 % reduction in classification accuracy) [3]. This is due to the influence of arm position on the muscular activation pattern when performing wrist/hand tasks [5, 7, 12]. This influence is also very relevant for the translation of myoelectric control algorithms to clinical prostheses and needs further investigation for regression methods aimed at the estimation of hand kinematics for simultaneous and proportional control mentioned above. Therefore, in this study, we investigate the effect of arm posture on the simultaneous and proportional myoelectric control over multiple DoFs of the hand/wrist in both able-bodied and amputee subjects. Compared to previous studies that limited this analysis to a finite number of classes sequentially classified and to able-bodied subjects only, the results of the current study will elucidate the effective impact of arm posture on the prosthetic users and on algorithms that allow a more advanced and intuitive control with respect to sequential on–off methods.

## Methods

### Subjects

Three individuals (2 males, 1 female; age range 31–42 years; referenced A1–A3) with unilateral trans-radial amputation participated in the experiment. All amputee subjects are users of conventional myoelectric prostheses, which articulate only one DoF. The information on the amputee subjects is summarized in Table 1.

**Table 1:** Summary information on the amputee subjects

Sub. ID	Age (years)	Time since amputation (years)	Position of amputation
A1	34	3	ca. 17 cm distal from elbow
A2	42	8	ca. 20 cm distal from elbow
A3	35	7	ca. 20 cm distal from elbow

In addition, 5 able-bodied subjects (2 males, 3 females; age range 24–40 years; all right-handed, referenced H1–H5) participated in the experiments. The eight subjects signed informed consent forms prior to their participation. The experimental protocol was approved by the local ethics committee of the Region North Jylland, Denmark.

### Experimental procedure

**Fig. 1:**
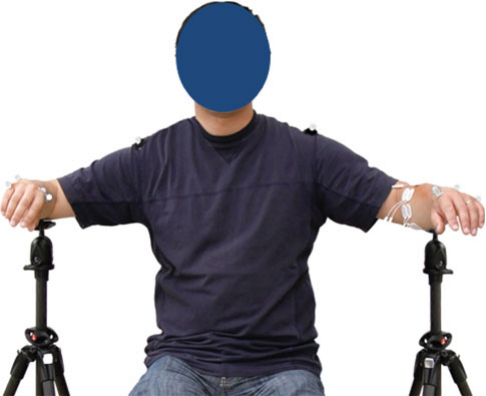
The experimental setup. The elbow supports for both sides were adjusted according to the three arm positions. In the position shown, the subject’s elbow is flexed 90° and armis abducted 75° from the torso (POS2)

The experimental setup was similar to the one described in [8]. During an experimental session, the subject sat in a custom-made chair, with the elbows resting on two armrests (Fig. 1). The elbow supports were adjusted so that the subject felt relaxed, and his/her shoulders and upper arms were in symmetric positions. Since each experimental session was long, the elbow supports were used so that the muscles of the upper limb (mainly the deltoid muscles) would not fatigue during the session. No support was provided to the forearm muscles during the experimental session. Eight cameras of an optical motion capture system (Qualisys AB, Gothenburg, Sweden) were placed in a circular pattern around the subject. The positions and orientation of the cameras were optimized to provide the maximum coverage of all markers during the intended movements.

During a recording session, the subjects were instructed to maintain their upper arms and forearms in 3 positions: POS1 with the elbows flexed at 90° and the arms 30° abducted from the torso; POS2 with the elbows flexed 90° and arms 75° abducted from the torso or to an extent comfortable for the subjects; and POS3 with the arms fully extended forward, perpendicular to the frontal plane. In each position, the subjects were asked to perform a series of hand movements that involved bilateral mirrored activations of the three DoFs of the wrists: flexion/extension (DoF1), radial/ulnar deviation (DoF2), and wrist pronation/supination (DoF3). The able-bodied subjects were instructed to perform mirror movements of their limbs while the amputee subjects were asked to imagine performing with the amputated limb the same movement performed with the intact limb. At the beginning of an experimental session, the subjects familiarized themselves with the protocol by performing the mirrored bilateral movements, with instruction from the experimenter. Then, the subject was instructed to perform two sets of movements for each arm position. In a particular continuous movement task (called a run), two or three DoFs were articulated concurrently. A total of seven runs were performed at each position (Table 2). The subjects were instructed to perform the runs at low to medium speed, which was subjectively controlled by the subject. The time it took to move from the neutral position to the maximal range of motion and come back to the neutral position was between 1 and 2 s. The maximal range of motion at each DoF was determined by the subject. The marker trajectories in the 3D space were visually inspected by the experimenter after each run. The run was repeated if the trajectories were deemed unsatisfactory due to excessive gaps in the acquired marker trajectories. Each run finished when the last complete movement (from neutral position to the maximal range of motion) was completed after 65 s, resulting in approximately 40–50 full repetitions of the movements. This duration was chosen to avoid fatigue and obtain a sufficient number of repetitions of the movements. Consecutive runs were separated by resting periods of 2–3 min, to avoid fatigue. The tested positions as well as the runs within a position were randomized for each subject.

**Table 2:** The descriptions of the seven runs of movements at each position

Set	Description	Active DoFs
1	Combined activation of two DoFs, in which one DoF was articulated sinusoidally, and the other was fixed at positions close to maximal range of motion	Run 1: DoF1 + DoF2
		Run 2: DoF2 + DoF1
		Run 3: DoF1 + DoF3
		Run 4: DoF3 + DoF1
		Run 5: DoF2 + DoF3
		Run 6: DoF3 + DoF2
2	Cyclic movements of DoF1 and DoF2, while alternating the direction of DoF3; unconstrained dynamic wrist movements	Run 7: DoF1 + DoF2 + DoF3

*Run* a continuous movement, *set* a group of runs, *DoF1* wrist flexion/extension, *DoF2* wrist radial/ulnar deviation, *DoF3* wirst pronatin/supination

### EMG recordings

Seven pairs of Ag–AgCl surface bipolar electrodes (Ambu NeuroLine 720) were placed on each forearm, with 23 mm inter-electrode distance. The electrode pairs were placed along the proximal/distal direction. At the intact side of the amputee subjects, the electrode pairs were placed around the thickest part of the forearm. This position is usually approximately 1/3 distal, measured from the olecranon process to the styloid process of the ulna. The electrode pairs were placed in a circle around the forearm with equal inter-pair distance, similarly to [14] and [8]. The first pair was placed approximately 1 cm medially from the ulnar bone and the remaining six pairs were positioned sequentially in the pronation direction. At the amputated side of the amputee subjects, the electrodes were placed on the same place as at the intact side, whenever possible. For the able-bodied subjects, the electrodes on both sides were placed as for the intact side of the amputee subjects. A reference armband (placed on one of the wrists for the able-bodied subjects and on the wrist of the intact side of the amputee subjects) was used as the common reference point. For improved line-interference rejection, all electrodes were connected via shielded cables to an EMG amplifier (EMG-USB, 128-channel, OT Bioelettronica), where the EMG signal was sampled at 2048 Hz and amplified at 2 k, with 12-bit AD resolution.

### Kinematics recordings

Passive-reflective markers (diameter 12 mm) were placed on both arms of the subjects. For the amputee subjects, seven markers were placed on the following anatomical landmarks at the intact side: one on the shoulder (prominent point of the Scapular Acromion); two at the elbow (prominent points of the medial and lateral epicondyle of humerus, denoted by MEP and LEP); two at the wrist (distal styloid processes of ulna and radius, denoted by STU and STR); and two at the hand (distal laterally and medially prominent points of the second and fifth metacarpal bone, denoted by RMC and UMC). At the amputated side, the first five markers were placed at the same place as the intact side, and the two additional markers were placed on the distal end of the stump, over the prominent points of the ulna and radius bones (found by palpation). For able-bodied subjects, seven markers were placed on both arms, at the same places as the intact limb of the amputee subjects. The 3D coordinates of the markers were acquired at 256 Hz, with error smaller than 0.5 mm (as indicated during the Qualisys calibration procedure). An external synchronization signal (20 Hz square wave, ±5 V) was provided to both the EMG acquisition system and the motion capture system so that the EMG traces and the kinematics could be synchronized offline.

### Data processing

The EMG and kinematics data were processed offline. The EMG signals were band pass filtered (10–450 Hz, second order Butterworth filter), and then resampled at 1,024 Hz. To estimate the kinematics at the wrist joint, the time domain (TD) feature set (mean absolute value, mean absolute value slope, zero crossings, and slope sign change) [6], and the 6-order autoregressive coefficients (AR) [4] (obtained by LMS linear prediction filter), namely the TDAR feature set, were used. For detailed information regarding these features, please refer to [12] and [13]. The analysis windows had duration of 100 ms and were overlapped by 60 ms.

**Fig. 2:**
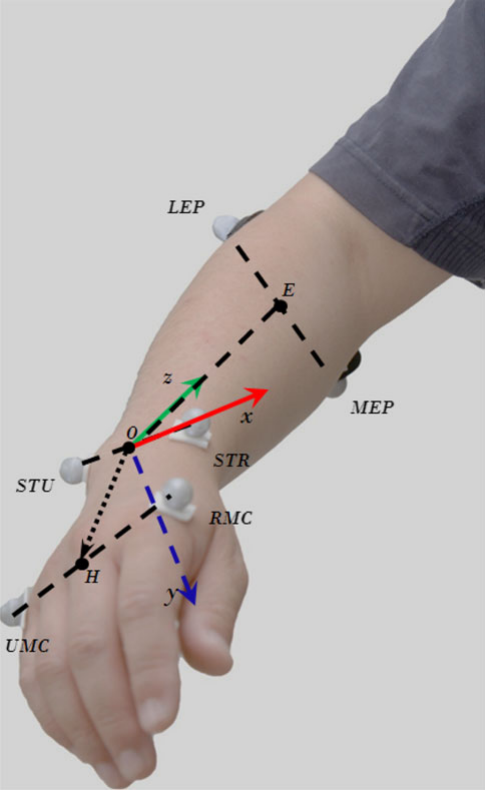
The positions of the markers and the coordinate system used to calculate the three joint angles

The angular displacements for each of the three DoFs were calculated from the coordinate system illustrated in Fig. 2. The origin of the system is at the center of the wrist, midway between the STR and STU, denoted by *O*. The *z*-axis set as the center axis of the forearm, positive in the proximal direction, pointing from *O* to *E*, the midway between MEP and LEP; the *y*-axis set as the dorsopalmar axis, positive in the anterior direction; and the *x*-axis set as the mediolateral axis of the wrist, positive in the lateral direction. Denoting the mid-point between RMC and UMC by *H*, and its projection on the three axes by *H*_*x*_, *H*_*y*_ and *H*_*z*_, the angles of the three DoFs are calculated by the following equations:

1 $$ \alpha_{1} = {\text{a tan}} \left( {\frac{{H_{y} }}{{H_{z} }}} \right) $$

2 $$ \alpha_{2} = {\text{a tan}} \left( {\frac{{H_{x} }}{{H_{z} }}} \right) $$

3 $$ \alpha_{3} = \measuredangle (\overset{\lower0.5em\hbox{$\smash{\scriptscriptstyle\rightharpoonup}$}} {\omega } ,\,\overset{\lower0.5em\hbox{$\smash{\scriptscriptstyle\rightharpoonup}$}} {l} ), $$

where $$ \overset{\lower0.5em\hbox{$\smash{\scriptscriptstyle\rightharpoonup}$}} {\omega } $$ is the vector from STU to STR, and $$ \overset{\lower0.5em\hbox{$\smash{\scriptscriptstyle\rightharpoonup}$}} {l} $$ is the vector from MEP to LEP. The range of *α*_1_ is ±90°, where a positive/negative angle indicates wrist flexion/extension. The range of *α*_2_ is ±90°, where a positive/negative angle indicates radial/ulnar deviation. The range of *α*_3_ is between 0° and 180°, where an angle greater/smaller than 90° indicates pronation/supination. The kinematic data were offline low-pass filtered (6 Hz, second order Butterworth filter). All kinematic data were re-sampled to 1,024 Hz, and synchronized with the corresponding EMG signal through the common synchronization signal.

Multi-layer perceptron (MLP) artificial neural networks were used to learn the association between the EMG features and the kinematic signals. The inputs to the MLPs were the EMG features, obtained using 100 ms long analysis windows with 60 ms overlap between two adjacent windows. The targets of the MLPs were the mean of the respective angle of the corresponding analysis windows. Three MLPs were used to estimate the three joint angles of the three DoFs. The number of neurons in the hidden layer was determined to be three, as reported in previous studies [8], where the number of EMG channels, EMG features and estimation target were similar to the current study.

#### Contralateral training of the MLPs

The MLP can be used to learn the wrist kinematics of one side (for example the intact side) from the EMG features from the other side (for example the amputated side), in both able-bodied [13] and amputee subjects [8]. This contralateral training approach for MLPs was used also in the current study. The inputs to the MLP were the EMG features from the amputated side for amputees or the dominant side for able-bodied subjects. The targets of the MLPs were the joint angles of the contralateral side calculated using Eqs. (1), (2), (3).

#### Effect of arm positions

The MLPs were trained using data from one arm position and tested in all positions. For each position, the randomly selected 4/5 of data of each run from that position were used for training data for the MLP. The rest 1/5 data from each run were used as testing data, resulting in a 5-fold cross-validation. All the data from the other two positions were also used as the testing data. The cases are referred to as intra-position when the training and testing data were from the same arm position and inter-position when the training and testing were from different arm positions.

#### Activated DoFs

Since the seven runs (Table 2) articulated different DoFs, four analysis scenarios based on the activated DoFs were considered. The analysis scenario referred to as *DoF12* indicates that only DoF1 and DoF2 were used for the analysis (runs 1 and 2). Similarly, the analysis scenario *DoF13* used only runs 3 and 4, and so on. When all the seven runs were used in the analysis, the scenario was called *DoF123*.

#### Effection of positional pooling

It has been shown in [3] that pooling data from different arm positions in the training phase significantly improved the classification accuracy for pattern recognition-based myoelectric control algorithms. The effect of this type of positional pooling was also investigated in the current study. For each fold of the 5-fold cross-validation, the training data from all three positions were pooled together to form the pooled training data for the current fold. MLPs with the same structure were used, and the testing data sets were the same as in the individual positional training schemes described above. The positional pooling analysis was done for all four analysis scenarios *DoF123*, *DoF12*, *DoF13*, and *DoF23*.

The performance of the MLPs of various configurations was measured by the multivariate *R* ^2^ index, proposed by [1]:

4 $$ R^{2} = 1 - \frac{{\sum\limits_{i = 1}^{D} {\sum\limits_{t = 0}^{{N_{i} }} {(\widehat{{\alpha_{i} (t)}} - \alpha_{i} (t))^{2} } } }}{{\sum\limits_{i = 1}^{D} {\sum\limits_{t = 0}^{{N_{i} }} {(\alpha_{i} (t) - \overline{{\alpha_{i} (t))}}^{2} } } }}. $$

where *D* is the number of DoFs considered, $N_{i}$ is the number of data points in the *i*th DoF, $\widehat{{\alpha_{i} (t)}}$ is the estimated angles, and $\overline{{\alpha_{i} (t)}}$ is the temporal mean of the measured angle, $\alpha_{i} (t)$. The numerator and the denominator of the fraction on the right-hand-side of (4) is the mean square error (MSE) of the estimation and the variance of the targets, respectively. This measure has shown to be less biased when the target angles have very small values, compared with the conventional MSE measure in estimation problems [1].

### Statistical analysis

As shown in the results section, the intra-position *R* ^2^ values were greater than those of the corresponding inter-position values. The main statistical analysis aimed at investigating the effect of arm position on the two subject groups, thus we eliminated from this analysis the variability due to the differences between the intra-position cases and inter-position cases. Two-way ANOVA was therefore performed on the *R* ^2^ values normalized with respect to the respective intra-position *R* ^2^ values. The analysis scenarios *DoF123*, *DoF12*, *DoF13*, and *DoF23* were analyzed by separate ANOVAs. Each ANOVA had two factors: testing arm positions (AP) and subject group (ST). The significance level was set to 95 %. The factor AP had three levels (three testing positions) and the factor ST had two levels (amputee and able-bodied subjects). For each of the four analysis scenarios, a full ANOVA with interaction was performed first. Since no statistically significant interactions between the two factors were found in all scenarios (see results), only the main effects were reported. Post hoc multiple comparisons (Tukey–Kramer) were performed when the main effects were significant.

## Results

**Fig. 3:**
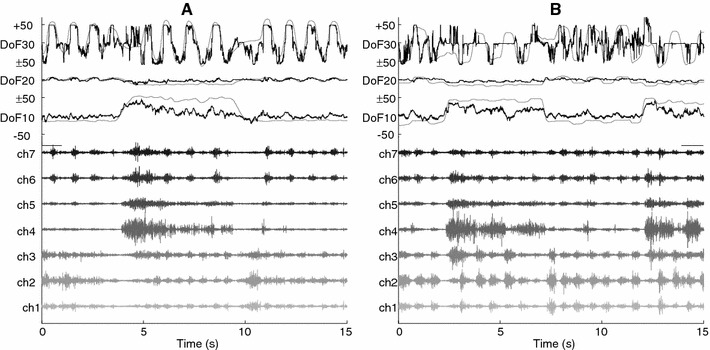
Representative EMG signals from an amputee subject (A2). In both panels, the measured joint angles (*thick dashed lines*) from the intact side are plotted against the estimated angles (*thin solid lines*). In the lower portion, the corresponding 7-channel EMG signals from the amputated side are shown, from which the estimated angles were obtained. In *panel A*, the arm position was POS1, and the ANN used was trained in the same position (an intra-position case) (*R* ^2^ = 72.4 %). In *panel B*, the arm position was POS3, the ANN used was the same as for *panel A* (an inter-position case) (*R* ^2^ = 56.2 %)

**Fig. 4:**
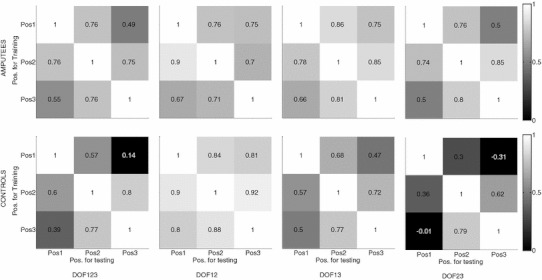
Mean *R* ^2^ values. The results from the amputee subjects are presented in the top row, and the results from the able-bodied subjects are in the bottom row. The four columns, from *left* to *right*, are the analysis scenarios *DoF123*, *DoF12*, *DoF13*, and *DoF23*. Because the baseline values (training and testing with data from the same position, the diagonal in each plot) varied significantly among the different scenarios, the *R* ^2^ values in each row were normalized with respect to the respective baseline values to illustrate the effect of arm position

Figure 3 shows a representative example of recorded EMG signals and estimated joint angles for an amputee (A2) performing movements that articulate the DoF1 and DoF3 (run 4) concurrently. The intra-position and inter-position *R* ^2^ values for the two subject groups are summarized in Table 3. In all cases, the intra-position *R* ^2^ values were significantly higher than the corresponding inter-position values (*p* < 10^−3^). This is likely due to the fact that muscle activities were indeed different when performing the same hand movements, while at different arm positions. The relative *R* ^2^ values of all analysis scenarios with respect to the respective intra-position values are summarized in Fig. 4.

**Table 3:** Summary of the *R* ^2^ values (mean ± sd)

		Intra-position (%)	Inter-position (%)
Amputees subjects	*DoF123*	61.3 ± 9.26	46.1 ± 16.8
	*DoF12*	66.6 ± 8.66	49.8 ± 16.4
	*DoF13*	74.8 ± 8.65	58.7 ± 14.7
	*DoF23*	76.6 ± 8.89	53.0 ± 17.2
Able-bodied subjects	*DoF123*	62.9 ± 6.87	34.0 ± 14.8
	*DoF12*	86.2 ± 5.38	73.9 ± 6.03
	*DoF13*	74.5 ± 8.16	48.0 ± 17.9
	*DoF23*	71.2 ± 6.22	39.9 ± 19.7

There was no interaction between the factors AP and ST. The results of subsequent ANOVA on the two main factors are summarized in Table 4. The testing position did not have a significant effect for *DoF12*, *DoF13*, and *DoF23*, and it was only significant for *DoF123*. These results means that there was no statistical difference of the relative *R* ^2^ values among the three testing positions, i.e., no one particular testing position resulted in a better performance than other testing positions. On the other hand, the subject group influenced the performance for *DoF12*, *DoF13*, and *DoF23*, but not for *DoF123*. Interestingly, when changing arm position, the amputee subjects had relatively higher performance (with respect to the respective baseline values) than intact-limb subjects in *DoF13* and *DoF*23 (rightmost column in Table 4). It is important to note that among the three DoFs, the third DoF (supination/pronation) is the most functional one for trans-radial amputees, followed by the first DoF (flexion/extension).

**Table 4:** The results of the ANOVA in all analysis scenarios

Analysis scenario	AP (arm pos.)	ST (sub. type)	Post hoc comp. for AP (arm pos.)	Post hoc comp. for ST (sub. type)
*DoF123*	*p* = 0.0283	*p* = 0.147	POS2 > POS1	n/a
*DoF12*	*p* = 0.256	*p* = 0.009	n/a	Amputee < control
*DoF13*	*p* = 0.91	*p* = 0.0259	n/a	Amputee > control
*DoF23*	*p* = 0.0869	*p* = 0.0088	n/a	Amputee > control

For post hoc comparison, only the significant comparisons are listed

**Fig. 5:**
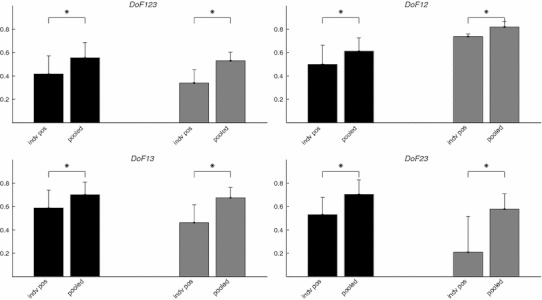
The effect of positional pooling in the four analysis scenarios. In each plot, the *dark* and *light bars* and the *vertical lines* are the mean and standard deviation of the *R* ^2^ values for amputee subjects, and able-bodied subjects, respectively. As shown in the figures, the positional pooling in MLP training significantly improved the estimation performance, measured by *R* ^2^ values. In all cases, one-tail paired *t* test showed that the *R* ^2^ values obtained when the MLPs were trained on pooled data were significantly higher than those obtained when the MLPs were trained on individual positions (*indicated by asterisks*). The significance level was 95 % for all tests

When the training data from all three positions were pooled together to form the pooled training sets, the *R* ^2^ values significantly improved, regardless of the training scenarios and subject group, as shown in Fig. 5. This improvement due to positional pooling is expected as the MLPs became positional aware with pooled training. This improvement is similar to positional pooling effect reported for pattern recognition-based myoelectric control algorithms [3].

## Discussion

We analyzed the effect of arm position on the performance of a direct joint kinematics estimation algorithm from surface EMG. The results showed that arm position does have a significant effect on the estimation performance for both trans-radial amputees and able-bodied control subjects. On average, the intra-position *R* ^2^ values were 61.3 and 62.9 %, for amputee and control subjects, respectively, and decreased to 46.1 and 34.0 % for the inter-position cases. This is due to the fact that the surface EMG characteristics of forearm muscles are influenced by the arm posture [5, 7, 12] and by the load at different directions [17]. These factors are relevant when the arm moves to different positions, or the support of the limb changes. Indeed, in a recent study [3] on able-bodied subjects, arm position was shown to significantly influence the performance of pattern classification-based myoelectric control algorithms. The results of the current study confirmed that such an effect is also relevant for algorithms based on the simultaneous and proportional control of multiple DoFs.

A further result of the current study is that, for the simultaneous and proportional control approach, arm position has a smaller influence for amputee subjects than for able-bodied subjects. The inter-position *R* ^2^ values (with respect to the intra-position values) of the amputee group were significantly greater than those of the able-bodied subject group in the *DoF13* and *DoF23* scenarios (Table 4). This difference might be due to the anatomical differences between the amputees and the able-bodied subjects. The forearm contains many muscles whose relative position may change considerably during dynamic movements, at least in normally limbed subjects. As a consequence, muscles can slide beneath the skin with respect to the electrodes when changing the arm position. This sliding may in turn alters the thickness of the biological tissue separating the muscles from the electrodes and thus, the recorded EMG patterns [11]. Due to the amputation, the remaining muscles are usually shorter in length, and fixed at the stump. As a consequence, there is much less variability in the muscle fiber length when the residual limb is in different positions. The fact that muscles or tendons are fixed in place to the bone in amputees may also reduce the possibility of relative shifting between muscles and electrodes, as well as changes in muscle geometry, which are more pronounced in normally limbed subjects. It is also worth noting that the drop in performance for able-bodied subjects when DoF3 was involved may be partly due to the fact that the EMG activity from the biceps muscle was not recorded. The *biceps brachii* is a powerful supinator especially when the forearm is flexed [2], as in the present experimental setup. However, this muscle was excluded to mimic a real life scenario, in which commercial hand prosthesis electrodes are mounted in the socket and thus record EMG signals only from the forearm muscles at the stump. The amputees may rely less than able-bodied subjects on the biceps activation because they are trained to use myoelectric prostheses with forearm muscle activity.

Another difference between amputees and able-bodied subjects when changing arm position is that the change in load due to gravity is not applied directly to the tendons of the muscles of amputees, contrary to able-bodied subjects. This could lead to lower gravity-compensatory muscle activities in amputees than for able-bodied subjects when arm posture is changed, with consequent less variability in EMG features and sensitivity of myoelectric control to arm position changes.

Despite the smaller influence for amputees, the arm position did have an effect on the kinematics estimation in both groups, so that this issue may be relevant in practical implementations. One way to reduce this effect is to include the data from different positions during the training of the ANN. A similar approach was investigated by [3] for pattern recognition-based algorithms, where the authors showed that when data from multiple positions were pooled together in the training phase of the classifier, the classification error was reduced significantly, as expected. In the current study, similar improvement due to positional pooling was also demonstrated. However, it would be impractical to include an excessive number of arm positions during the experimental (training) protocols. Therefore, this problem remains currently relevant and without a practically applicable robust solution.

In conclusion, this study showed that arm position has an influence on the accuracy of kinematics estimation from EMG in both able-bodied and amputee subjects. Interestingly, this effect was less pronounced for amputees, thus the impact of this issue in practical implementations may be less important than expected when analyzing able-bodied subjects only. Nevertheless, algorithms that deal with the influence of arm posture in myoelectric control are needed for clinical applications.
